# 

**Published:** 1857-09

**Authors:** 


### DENTAL REGISTER

### OF THE WEST.

EDITED BY

J. TAFT AND GEO. WATT.

September, 1857.

Published Quarterly at $3 Per Annum.

CINCINNATI:

WRIGHTSON & CO., PRINTERS.

1857.

